# Identification of Promiscuous African Swine Fever Virus T-Cell Determinants Using a Multiple Technical Approach

**DOI:** 10.3390/vaccines9010029

**Published:** 2021-01-07

**Authors:** Laia Bosch-Camós, Elisabet López, María Jesús Navas, Sonia Pina-Pedrero, Francesc Accensi, Florencia Correa-Fiz, Chankyu Park, Montserrat Carrascal, Javier Domínguez, Maria Luisa Salas, Veljko Nikolin, Javier Collado, Fernando Rodríguez

**Affiliations:** 1 IRTA, Centre de Recerca en Sanitat Animal (CReSA, IRTA), Campus de la Universitat Autònoma de Barcelona, 08193 Bellaterra, Spain; laia.bosch@irta.cat (L.B.-C.); elisabeth.lopezf@gmail.com (E.L.); mariajesus.navas@irta.cat (M.J.N.); sonia.pina@irta.cat (S.P.-P.); flor.correa@irta.cat (F.C.-F.); 2 OIE Collaborating Centre for the Research and Control of Emerging and Re-Emerging Swine Diseases in Europe (IRTA-CReSA), 08193 Bellaterra, Spain; francesc.accensi@irta.cat; 3 UAB, Centre de Recerca en Sanitat Animal (CReSA, IRTA-UAB), Campus de la Universitat Autònoma de Barcelona, 08193 Bellaterra, Spain; 4 Departament de Sanitat i Anatomia Animals, Facultat de Veterinària, UAB, 08193 Bellaterra, Spain; 5 Department of Stem Cells and Regenerative Biology, Konkuk University, Seoul 05029, Korea; chankyu@konkuk.ac.kr; 6 Instituto de Investigaciones Biomédicas de Barcelona-Unidad de Espectrometría de Masas Biológica y Proteómica, Consejo Superior de Investigaciones Científicas (CSIC), 08193 Bellaterra, Spain; montserrat.carrascal.csic@uab.cat; 7 Departamento de Biotecnología, Instituto Nacional de Investigación y Tecnología Agraria y Alimentaria (INIA), 28049 Madrid, Spain; juncal@inia.es; 8 Centro de Biología Molecular Severo Ochoa, Consejo Superior de Investigaciones Científicas and Universidad Autònoma de Madrid, 28049 Madrid, Spain; mlsalas@cbm.csic.es; 9 Boehringer Ingelheim Veterinary Research Center (BIVRC) GmbH & Co. KG, 30559 Hannover, Germany; veljko.nikolin@boehringer-ingelheim.com; 10 Departament de Biologia Cel·lular, Fisiologia i Immunologia, Campus de la Universitat Autònoma de Barcelona, 08193 Bellaterra, Spain; javiercolladomiguens@gmail.com

**Keywords:** ASFV, CD8^+^ T-cells, antigen presentation, IFNγ ELISpot, epitope predictions, immunopeptidomics, promiscuous epitope

## Abstract

The development of subunit vaccines against African swine fever (ASF) is mainly hindered by the lack of knowledge regarding the specific ASF virus (ASFV) antigens involved in protection. As a good example, the identity of ASFV-specific CD8^+^ T-cell determinants remains largely unknown, despite their protective role being established a long time ago. Aiming to identify them, we implemented the IFNγ ELISpot as readout assay, using as effector cells peripheral blood mononuclear cells (PBMCs) from pigs surviving experimental challenge with Georgia2007/1. As stimuli for the ELISpot, ASFV-specific peptides or full-length proteins identified by three complementary strategies were used. In silico prediction of specific CD8^+^ T-cell epitopes allowed identifying a 19-mer peptide from MGF100-1L, as frequently recognized by surviving pigs. Complementarily, the repertoire of SLA I-bound peptides identified in ASFV-infected porcine alveolar macrophages (PAMs), allowed the characterization of five additional SLA I-restricted ASFV-specific epitopes. Finally, in vitro stimulation studies using fibroblasts transfected with plasmids encoding full-length ASFV proteins, led to the identification of MGF505-7R, A238L and MGF100-1L as promiscuously recognized antigens. Interestingly, each one of these proteins contain individual peptides recognized by surviving pigs. Identification of the same ASFV determinants by means of such different approaches reinforce the results presented here.

## 1. Introduction

African swine fever (ASF) is a hemorrhagic viral disease of pigs that courses with lethality rates up to 100% in its acute forms. Due to the devastating impact of the disease, including national and international trading restrictions, ASF is included in the Terrestrial Animal Health Code of the World Organization for Animal Health (OIE) as a notifiable disease. To date, no commercial vaccines are available against ASF, and hence, control strategies are based on early diagnosis, animal quarantine and slaughter of infected and in contact pigs. Despite guidelines have proven effective in wealthy areas where compensation policies are correctly applied [1], their implementation in less favored areas have demonstrated inefficient, recommending additional efforts in research and development of complementary antiviral treatments and vaccines, not available today.

African swine fever virus (ASFV) is the sole member of the family *Asfarviridae*, genus *Asfivirus*, and it is included in the nucleocytoplasmic large DNA virus superfamily [2]. ASFV was first described in Kenia in 1921 as an endemic virus continuously circulating between African wild pigs and ticks from the *Ornithodoros* genus in an asymptomatic manner [3]. Since then, ASF remained endemic in many sub-Saharan countries with sporadic exportations to other continents. Two ASFV entries in Portugal, dated in 1957 and 1960, provoked 40 years of ASFV endemicity in the Iberian peninsula, the sporadic occurrence of ASF in some countries of Europe and South America and the establishment of ASFV in Sardinia since 1978 until today [4]. Continental Europe became free of ASF in 1997, but only 10 years later, in 2007, ASFV reentered Europe through Georgia, rapidly expanding to neighboring countries of Eastern Europe [5]. In 2014, the virus entered the European Union (EU) territory for the first time, affecting both domestic pigs and wild boars, the latter playing a critical role in ASF spread. In this area, the main causes of ASFV transmission include pig to pig contact, infected pig products, or infected fomites, such as transport vehicles [4]. Conversely, wild boar-mediated transmission has been considered a minor risk factor in Asia, albeit this view is currently being revised, with some countries reporting relevant outbreaks in their wild boar populations [6]. Since its first declaration in China in 2018, most probably due to the importation of contaminated pork products, ASFV has expanded extremely fast to all neighboring countries, reaching more than 28 countries from Asia and Oceania, causing an economic crisis of gigantic proportions [7,8]. Therefore, developing safe and efficacious vaccines against ASF is a priority for the swine industry worldwide [9].

Immunization with recombinant live attenuated viruses (LAV) conferred protection against experimental challenge with genotype II ASFV strains, currently circulating in Europe and Asia [10,11,12,13,14]. Unfortunately, the molecular and immunological mechanisms eliciting this immunity are poorly understood, albeit innate immune responses [15,16,17,18], and both ASFV-specific antibodies [19,20] and CD8^+^ T-cells [21], may play complementary roles. CD8^+^ T-cell responses, in the absence of antibodies, have demonstrated to be responsible for the partial protection triggered by DNA vaccines in the absence of antibodies [22,23]. Nevertheless, the protection afforded so far has been limited to homologous lethal challenge with E75 (genotype I) [22,23], and has proved unsuccessful against experimental challenge with Georgia2007/1 [24]. In addition, the complexity of ASFV, encoding more than 150 proteins [25,26,27,28], challenges the identification of the specific antigens and determinants inducing protective responses.

The aim of this study was to explore the effectiveness of three different strategies to identify ASFV CD8^+^ T-cell epitopes and ASFV proteins, presented in the SLA I-context and promiscuously recognized by CD8^+^ T-cells from ASF survivors. The detection of ASFV-specific T-cells was assessed by IFNγ ELISpot, using peripheral blood mononuclear cells (PBMCs) as effector cells from pigs experimentally vaccinated with BA71ΔCD2 [12] and surviving the infection with Geogia2007/1, the virulent ASFV globally circulating. Different stimuli were used for the ELISpot assay: (i) synthetic peptides selected by in silico predictions; (ii) synthetic peptides selected by immunopeptidomics; or (iii) autologous fibroblasts transfected with plasmids encoding individual full-length open reading frames (ORFs) fused to ubiquitin [22,23,29,30]. Together with a complete list of ASFV peptides susceptible to be presented in the SLA I context, here, we report a collection of specific peptides and proteins that are specifically recognized by T-cells from ASF surviving pigs. Furthermore, the three ASFV antigens characterized as promiscuously inducing specific T-cell responses (independently of the SLA I haplotype), were concomitantly identified by the different methods here implemented.

## 2. Materials and Methods

### 2.1. Cells and Viruses

Porcine alveolar macrophages (PAMs) from healthy conventional pigs (Landrace × Large White) were obtained by bronchoalveolar lung lavage. Porcine PBMCs were isolated from whole blood using Histopaque-1077 density gradient solution (Sigma-Aldrich, Saint Louis, MO, USA). Porcine primary fibroblast cultures were obtained from 2 cm^2^ pieces of ear tissue sample following previously described protocols [31].

Two ASF viruses were used: BA71 and the live attenuated BA71ΔCD2 virus, a deletion mutant from BA71 lacking the CD2v gene (EP402R) previously obtained in the laboratory [12].

### 2.2. Multiparametric In Silico Predictions of ASFV-CD8^+^ T-Cell Epitopes

Georgia2007/1 proteome was retrieved from Uniprot (UP000141072) for in silico CD8^+^ T-cell epitope prediction. Predictions were made using the NetMHCpan 3.0 software [32], considering 42 SLA class I alleles. Peptides ranging from 8 to 11 amino acid residues, with an IC50 (concentration of peptide inhibiting binding of a standard peptide by 50%) below 500 nM were selected. A total of 8648 different sequences were obtained. To further select the most promising theoretical CTL candidates, additional features were considered for each peptide, including: (i) proteasome cleavage, analyzed by using the MHC-I Processing tool from IEDB (http://tools.iedb.org/processing); (ii) promiscuity: the number of SLA I alleles predicted to bind the peptide with an affinity of 500 nM or lower (out of the 42 alleles screened); (iii) overlap: the number of predicted peptides with a SLA binding affinity of 500 nM or lower, overlapping in at least one amino acid to a given polypeptide; (iv) peptide immunogenicity [33]; and (v) binding affinity of peptides to the transporter associated with antigen processing (TAP) using TAPREG scoring [34], successfully used in the laboratory to identify two CD2v CTL peptides from the E75 ASFV strain [22]. The list of candidates (266 peptides) was obtained, taking into account all these parameters. Finally, 64 larger peptides (15–27 amino acids) were added to the peptide collection, according exclusively to the presence of ten or more overlapping peptides within their sequence, being considered putative hot spots for promiscuous SLA I antigen presentation.

### 2.3. Typing of SLA I Genes

Typing PAMs were performed using genomic DNA isolated using NucleoSpin Blood kit (Macherey-Nagel, Düren, Germany), and *SLA-1*, *SLA-2* and *SLA-3* classical SLA I genes. Typing was performed following previously established protocols [35,36,37].

### 2.4. In Vitro Infection of PAMs with ASFV

For each virus, a 6-well plate was used with 5 × 10^6^ PAMs/well. PAMs were infected using a multiplicity of infection (MOI) of 0.01. Cells were harvested by scrapping and pellets frozen at −80 °C until used. Cell supernatants were harvested at different times to follow the virus kinetics by qPCR as previously described [23].

### 2.5. Determination of Cell Viability and Percentage of Infected Cells by Flow Cytometry

Cell viability was measured using the LIVE/DEAD fixable violet dead cell stain kit (Invitrogen, Carlsbad, CA, USA). To determine the percentage of infected cells, PAMs were permeabilized using Cytofix/Cytoperm fixation/permeabilization kit (BD Biosciences, Allschwil, Switzerland). Anti-ASFV p72 mouse monoclonal antibody (clone 1BC11, Ingenasa, Madrid, Spain) diluted 1/100 was used, followed by APC-conjugated AffiniPure Goat Anti-Mouse IgG1 (Jackson ImmunoResearch, West Grove, PA, USA) diluted 1/200 as secondary antibody. A BD FACSAria IIu was used for analysis (BD Biosciences).

### 2.6. Affinity Purification of SLA I Molecules

Anti-SLA I monoclonal antibody (mAb) 4B7/8 [38] was purified from hybridoma supernatant by affinity chromatography using a Sepharose 4Fast Flow resin, coupled with an antibody recognizing mouse kappa L chains (Capture Select LCkappa (mur), Thermofisher, Waltham, MA, USA). The antibody was then extensively dialyzed against 0.1 M sodium carbonate buffer pH 8.3 containing 0.5 M NaCl in a D-tube dialyzer maxi with a molecular weight cut-off of 12–14 kDa (Novagen, Madison, WI, USA). Dialyzed α-SLA I antibody was next coupled to CNBr Sepharose beads, following the manufacturer’s instructions (GE Healthcare, Chicago, IL, USA). PBS 0.1% (*w*/*v*) sodium azide was used for long-term storage of the coupled sepharose at 4 °C.

Cell pellets were thawed on ice and lysed with 500 µL of 1% n-Dodecyl β-D-Maltoside (Thermo Fisher Scientific, Waltham, MA, USA) in immunoprecipitation buffer (50 mM Tris-HCl, pH 8 containing 150 mM NaCl) plus 1X complete protease inhibitor cocktail (Thermo Fisher Scientific, Waltham, MA, USA), and incubated with an equal volume of the sepharose-conjugated α-SLA I for 8 h at 4 °C with end-over-end rotation. Non-specifically bound molecules were removed by washing and, finally, SLA I-peptides complexes were eluted in 4–5 sepharose volumes of 50% acetonitrile, 5% formic acid, and stored at −80 °C until further analysis.

### 2.7. Western Blot to Detect Immunoprecipitated SLA I-Peptide Complexes

A total of 2.5% of each sample was run in a 4–12% gradient NuPAGE Bis-Tris acrylamide SDS-PAGE (Invitrogen, Carlsbad, CA, USA). His-tagged protein ladder (Thermofisher, Waltham, MA, USA) was used as molecular weight marker. The gel was transferred to a nitrocellulose membrane (Amersham, Protran Premium), using a XCell SureLock™ Mini-Cell with a blot module (Thermofisher, Waltham, MA, USA). Following transfer, the nitrocellulose membrane was blocked in 3% non-fat milk (*w*/*v*). 4B7/8 α-SLA I hybridoma supernatant (4 µg/mL) and anti-mouse IgG HRP-conjugated (Sigma-Aldrich, Saint Louis, MO, USA, 1:10,000) were used as primary and secondary antibodies, respectively. For the His-tag marker, mouse anti-His tag HRP-conjugated (Novex, Thermofisher, Waltham, MA, USA) 1:100,000 was used. After extensive washing, the specific signal on the membrane was developed by using Western Lightning Ultra chemiluminescence substrate (PerkinElmer, Waltham, MA, USA) for 5 min at RT in the dark. A Fluorchem HD2 (Alpha Innotech, Kasendorf, Germany) was used for imaging.

### 2.8. On-Tip Desalting and LC-MS/MS Analysis

The proteomic analysis was performed in the CSIC/UAB Proteomics Facility of IIBB-CSIC that belongs to ProteoRed, PRB2-ISCIII, supported by grant PT13/0001. Samples were desalted with TopTips C18 (PolyLC Inc., Columbia, MD, USA), following the standard procedure. The eluates obtained from the desalting process were evaporated to dryness and reconstituted in 20 µl of 5% MeOH, 1% HCOOH for analysis by LC-MS/MS (at the Proteomics Laboratory of CSIC-UAB). The MS system used was an LTQ XL Orbitrap (ThermoFisher) equipped with a nanoESI ion source. The total amount of each sample (20 µL) was loaded into the chromatographic system consisting in a C18 preconcentration cartridge (Agilent Technologies, Barcelona, Spain) connected to a 15 cm long, 100 µm i.d. C18 column (Nikkyo Technos Co Ltd., Tokyo, Japan). The separation was done at 0.4 µL/min in a 120-min acetonitrile gradient from 3 to 40% (solvent A: 0.1% formic acid, solvent B: acetonitrile 0.1% formic acid). The HPLC system was composed of an Agilent 1200 capillary nano pump, a binary pump, a thermostated micro injector and a micro switch valve. The LTQ XL Orbitrap was operated in the positive ion mode with a spray voltage of 1.8 kV. The spectrometric analysis was performed in a data dependent mode, acquiring a full scan followed by 10 MS/MS scans of the 10 most intense signals detected in the MS scan from the global list. The full MS (range 400–1800) was acquired in the Orbitrap with a resolution of 60,000. The MS/MS spectra were done in the linear ion-trap.

### 2.9. Database Search and Peptide Identification

All LC-MS/MS spectra were searched using SEQUEST (Proteome Discoverer v1.4, ThermoFisher, Waltham, MA, USA) using a combined database, including *Sus Scrofa*, BA71 and Georgia2007/1 ASFV genomes, and the 6-frame translation of each virus genome (in order to identify peptides in and out of known ORFs). The following parameters were fixed: peptide confidence = High, peptide rank = 1, Xcorr > 2.

### 2.10. PBMC Purification

Male Landrace × Large White piglets aged 6–8 weeks old were used, and animal care and procedures were carried out in accordance with the guidelines of the Good Experimental Practices and under the supervision of the Commission of Animal Experimentation of Generalitat de Catalunya (approval code CEA-OH/9212/2). In vivo experiments were performed at the biosafety level 3 facilities at the Centre de Recerca en Sanitat Animal (IRTA-CReSA, Barcelona, Spain).

Pigs were intramuscularly immunized with BA71ΔCD2 (3.3 × 10^4^ or 10^6^ PFU), and three weeks after, pigs were challenged with 10^3^ GEC of Georgia2007/1. Three to four weeks later, PBMCs were collected, at the peak of ASFV-specific humoral and T-cell responses [12]. PBS immunized pigs are always used as controls for the assays, succumbing the lethal Georgia2007/1 infection between days 5 and 10 with acute ASF-clinical signs and high viral loads in serum. As previously described, BA71∆CD2-immunized pigs survive Georgia2007/1 challenge with little or no ASF-compatible signs and without significant viral load. From three weeks post-challenge, immunized pigs were negative for ASFV in serum and positive for ASFV specific responses, including specific antibodies and T-cells (detectable from 14 days post-immunization).

### 2.11. Porcine IFNγ ELISpot

IFNγ response was assessed by ELISpot assay using purified mouse anti-pig IFNγ Clone P2G10 (BD Pharmingen, Allschwil, Switzerland) as capture antibody and biotinylated mouse anti-porcine IFNγ antibody P2C11 (BD Pharmingen, Allschwil, Switzerland) as detection antibody, following a previously reported method [23]. ASFV-specific peptides (4 µg/mL) or BA71ΔCD2 (10^6^ PFU/mL) were added as specific stimuli and PBMCs were incubated o/n at 37 °C. All peptides were synthesized by ProImmune Ltd. (Oxford, UK) to >85% purity. RPMI and 10 µg/mL phytohemagglutinin-M (PHA-M, Sigma-Aldrich, Saint Louis, MO, USA) were used as negative and positive controls, respectively.

Transfected fibroblasts were used as APCs at a 1:5 ratio with autologous PBMCs. The ASFV gene expression library available in our laboratory at the time consisted in 73 plasmids encoding individual ORFs from the E75 ASFV isolate (GenBank accession number FN557520.1), cloned in frame with ubiquitin into the pCMV vector [29]. Plasmid transfection of the fibroblasts was done by electroporation (pulse voltage = 1700 V, pulse width = 20 ms, pulse number = 1), using the Neon Transfection System 10 µL Kit (Invitrogen, Carlsbad, CA, USA). Fibroblasts electroporated with the empty pCMV-Ub plasmid were used as a negative control for the assay. Finally, electroporated cells were placed in the corresponding well of a 96-well plate with the autologous PBMCs and proceeded as described above.

## 3. Results

### 3.1. In Silico Prediction of CD8^+^ T-Cell Epitopes Using the Georgia2007/1 Proteome and In Vitro Validation as ASFV-Specific T-Cell Epitopes

Covering the entire proteome Georgia2007/1 with overlapping 8–11-mer peptides would need synthesizing more than 50,000 peptides, which was out of the scope of this project. In order to reduce the number of peptides to synthesize, the Georgia2007/1 proteome was first screened with the NetMHCpan3.0; thus, selecting a total of 8648 peptides ranging from 8 to 11 amino acid residues, with optimal SLA I binding properties (IC50 < 500 nM). The theoretically best fitting peptides were finally classified attending to multiple parameters: TAP-binding affinity, proteasome cleavage, promiscuity, overlapping, and peptide immunogenicity. The combination list was reduced to the best 266 candidates (Supplementary Table S1), which, together with 64 longer sequences (12–27 amino acids in length), selected exclusively according to the presence of multiple overlapping predicted epitopes (Supplementary Table S2), were individually synthesized. The 330 selected peptides belonged to 110 different proteins (from the 166 of Georgia2007/1 proteome). Interestingly, 50% of the selected peptides belonged to 22 single proteins, half of them (53.3%) were described as late proteins and a large proportion (22.2%), accounting for enzymes involved in nucleic acid metabolism (Supplementary Table S3).

After chemical synthesis, only one of the 330 predicted peptides was capable of stimulating a specific IFNγ response when using PBMCs from BA71ΔCD2-immunized pigs before and after surviving Georgia2007/1 challenge. The identified peptide corresponded to residues 68–86 of the MGF100–1L, and was specifically recognized by 11 out of the 20 (55%) pigs tested. It is worth noting that the identified sequence corresponded to a 19-mer peptide containing at least nine predicted CD8^+^ T-cell overlapping epitopes (Table 1).

### 3.2. Identification of SLA I-Restricted Peptides by Mass Spectrometry-Based Immunopeptidomics and In Vitro Validation as ASFV-Specific T-Cell Epitopes

**Figure 1:**
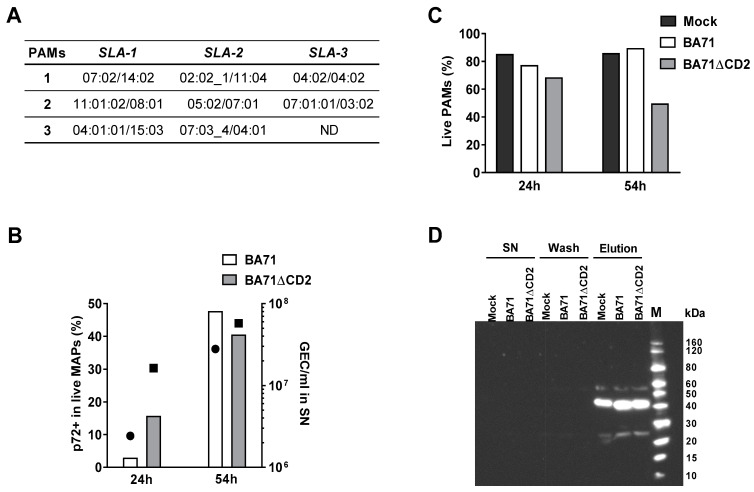
(**A**) *SLA-1*, *SLA-2* and *SLA-3* alleles for the three individual pigs from which PAMs were extracted and used in the immunopeptidomics studies. Allele annotation from the IPD-MHC Database. ND: not determined due to low quality of the DNA sample; (**B**) percentage of ASFV-infected PAMs from a representative sample at 24 hpi and 54 hpi, as detected by flow cytometry using anti-p72 monoclonal antibody (bars) and the corresponding ASFV detection by qPCR in supernatants (circles for BA71 and squares for BA71ΔCD2); (**C**) percentage of live PAMs from a representative sample at 24 hpi and 54 hpi detected by VIVID staining; (**D**) Western blot for the detection of SLA I molecules in supernatants (SN) of infected cell lysates after incubation with anti-SLA I-coupled sepharose beads, in the last sepharose wash before elution (Wash), or in the eluted SLA I-peptide complexes (Elution). M: molecular weight marker.

To perform the mass spectrometry (MS)-based immunopeptidomic analysis, peripheral alveolar macrophages (PAMs) from three pigs with different SLA I haplotypes (Figure 1A), were individually infected with the virulent BA71 or with the attenuated BA71ΔCD2 ASFV strain, at a MOI of 0.01. Cells and cell supernatants were obtained at 24 and 54 h post-infection (hpi), and the kinetics of both the percentage of infected live cells and virus production were compared (Figure 1B). As expected, the maximum values were obtained at 54 hpi, being similar for both viruses, despite the cell death rate seeming higher for BA71ΔCD2 than for BA71 infected cells (Figure 1C). Cell extracts obtained at this time point were selected for further proteomic analysis. After anti-SLA I immunoprecipitation and elution, the presence of SLA I-peptide complexes was confirmed by Western blot using an anti-SLA I antibody (Figure 1D). The intense band observed corresponds to the 45 kDa SLA class I heavy chain, while the two lighter bands correspond to traces of the heavy and light chains of the anti-SLA I antibody used for the immunoprecipitation detached from the sepharose beads (see Supplementary Figure S1, for a complete view of the WB).

BA71-infected PAMs led to the determination of 50 ASFV sequences, while 82 peptides were profiled from the BA71ΔCD2-infected samples (Table 2, Supplementary Table S4), despite the percentage of live cells recovered at 54 hpi is lower for the later (Figure 1B), and both the percentage of infected cells and virus titers obtained from supernatants are comparable (Figure 1C). Thus, in total, 132 SLA I-bound peptides were identified, corresponding to 106 different ASFV sequences that belonged to 56 different ASFV proteins (Table 2). Interestingly, 91 (85.8%) of the 106 sequences identified matched identical sequences in Georgia2007/1, and 13 of the peptides (12.3%) differed only in one or two amino acids that theoretically did not play key roles in SLA I binding. Finally, only two (1.9%) of the eluted peptides showed significant divergence with the Georgia2007/1 sequence. The differences observed between BA71 and BA71ΔCD2 PAM extracts did not only affect the number of peptides identified, but also the number of different proteins to which the peptides belong. From the 56 proteins identified, only 21 were commonly represented in the peptide-elution pools obtained from BA71 and BA71ΔCD2 PAM extracts, while seven and 28 were uniquely identified after BA71 and BA71ΔCD2 infection, respectively (Table 2).

As expected for optimal SLA I ligands, 50% of the eluted peptides were 9-mers. A total of 35.6% of them corresponded to proteins with unknown function, albeit proteins involved in transcription and replication, morphogenesis, host cell interaction and from multigene families were also identified. As described for the in silico prediction, a large proportion of the eluted peptides mapped within late proteins (62.2%) (Table 2), albeit it is worth mentioning that none of them matched the in silico predicted ones. The ASFV protein from which the larger number of peptides were identified was the uncharacterized B475L, with a total of nine peptides (six different sequences), followed by the putative helicase encoded by the *D1133L* ORF [39] and the uncharacterized K145R protein, from which seven (five different sequences) and six peptides (three different sequences) were identified, respectively. Five peptides were identified from the structural protein p37 (pp220 product) encoded by the *CP2475L* gene [40], the major capsid protein B646L and the uncharacterized protein M1249L, corresponding to five, four and three different sequences, respectively (Table 2).

Finally, it is worth mentioning the identification of five additional SLA I peptides mapping out of any known ORF (Supplementary Table S4, all detected from BA71 CD2-infected samples. Homologous sequences of four out of these five peptides were found in the genome of the Georgia2007/1.

Out of the 111 different peptides identified by the immunopeptidomics approach, five (4.5% of the total), were recognized by ASFV-specific T-cells. Thus, in vitro stimulation of PBMCs collected from BA71∆CD2-immunized pigs surviving Georgia2007/1 challenge with these individual peptides, specifically induced IFNγ secretion detectable by ELISpot (Table 3).

Aiming to confirm the phenotype of the peptide-specific T-cells, PBMCs from a BA71∆CD2-immunized pig or from a non-immunized pig (control) were in vitro stimulated with a mix of the positively identified peptides (Table 3) in the presence of Brefeldin A, to allow for the intracellular accumulation of IFNγ (Supplementary Figure S2). PBMCs were finally fluorescence-labelled with specific surface markers and intracellularly, with an anti-IFNγ antibody (see details in Figure S2 legend). ASFV-specific peptides specifically stimulated single positive CD8^+^ T-cells, exclusively detected in the BA71∆CD2-immunized pig and not in the control pigs (Figure S2). As expected, IFNγ^+^ CD8α^high^ T-lymphocytes, were also detected when stimulating the PBMCs with BA71∆CD2, although the highest proportion of IFNγ^+^ T cells were, in this case observed in CD4^+^/CD8^+^ T-cells, fitting with activated and effector memory phenotype in swine [41,42]. 

Two of the identified peptides belong to early expressed proteins: MGF505-7R and A238L or IkB-like protein, involved in transcription inhibition [43]. The other three peptides belong to late (K145R and H339R) or early/late (I226R) proteins with unknown functions. Interestingly, only the K145R peptide was identified in both BA71 and BA71∆CD2-infected PAM extracts. Coincidently, this K145R peptide, together with the A238L and the MGF505-7R peptides, exclusively identified in BA71∆CD2-cell extracts, were recognized by at least 20% of the tested animals. Conversely, the H339R and the I226R exclusively identified in BA71-infected samples were specifically recognized by only one out of the ten pigs tested (Table 3). Far from being conclusive, these results indicate that the peptide repertoires presented by BA71ΔCD2 and BA71 are not only quantitative, but also qualitatively different.

### 3.3. Identification of ASFV Full-Length Proteins Promiscuously Recognized by ASFV-Specific T-Cells

Complementary to the identification of ASFV specific CD8^+^ T-cell epitopes by in silico predictions and peptide-elution experiments, a third approach was followed aiming to identify ASFV full-length proteins promiscuously recognized by ASFV specific T-cells. Instead of using synthetic peptides as specific stimuli in our ELISpot assay, autologous pig fibroblasts (from the same pig than the PBMCs used as effector cells), were used as antigen presenting cells (APCs), after being transfected with plasmids encoding 73 individual full-length proteins (Table 4).

**Figure 2:**
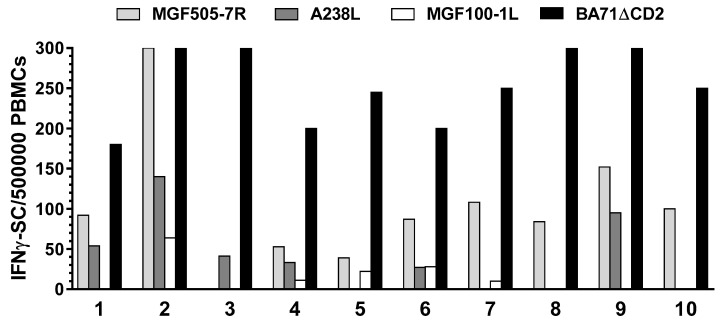
IFNγ response assessed by ELISpot assay using fibroblasts transfected with the pCMV-Ub-MGF505-7R, pCMV-Ub-A238L and pCMV-Ub-MGF100-1L plasmids as APCs, and PBMCs from ASF-convalescent animals as effector cells (pig individual numbers on the x axis). Values represented correspond to the specific IFNγ-SC (spots) found after stimulation with autologous fibroblasts transfected with each specific plasmids, once subtracted the number of spots found with pCMV-Ub (empty plasmid) transfected fibroblasts (always <10). Levels of specific IFNγ-SC observed after stimulation with the BA71ΔCD2 virus are also shown.

Each protein was expressed as a fusion with ubiquitin to improve their SLA I presentation and their recognition by the specific T-cells [22,23]. PBMCs (matching the APCs) from BA71ΔCD2-immunized pigs that survived the Georgia2007/01 challenge, were used again as effector cells in an IFNγ ELISpot assay. Seven pools of 10 to 11 plasmids (Table 4) were initially electroporated into fibroblasts and then used to specifically stimulate the production of IFNγ by the specific T-cells at a ratio of 1:5 (APC:PBMCs). Finally, plasmids from the pools capable of specifically stimulating IFNγ responses were individually tested. This screening led to the identification of three full-length proteins (from the 73 tested) recognized by ASFV-specific T-cells: MGF505-7R, MGF100-1L and A238L (Figure 2). Interestingly, all three ASFV antigens were broadly recognized by PBMCs from at least 50% of the pigs tested. Thus, a single clone, pCMV-Ub-MGF505-7R, was capable to stimulate specific IFNγ responses in all but one of the ten animals tested (Figure 2). The two additional antigenic proteins identified using this methodology: A238L and MGF100-1L, despite showing less promiscuity than MGF505-7R, were still broadly recognized by ASFV-specific T cells in five and six out of the 10 pigs tested (Figure 2).

Interestingly, peptides from these three proteins were previously identified as ASFV-specific T-cell epitopes, two from the immunopeptidomics assays using PAMs infected with BA71ΔCD2 (MGF505-7R_334–341_ and A238L_81–91_) and one from the in silico predictions (MGF100-1L_68–86_), validating our methodologies. The specific peptides were always recognized by a lower proportion of ASF-surviving pigs (20–30%) than the full-length proteins (50–90%), most probably reflecting their SLA I-restricted nature. These results strongly suggest the presence of multiple ASFV-specific T-cell epitopes in these full-length proteins, and argue in favor of their use in future experimental vaccination studies.

## 4. Discussion

Availability of effective vaccines against ASF would greatly improve the management of the disease as well as eradication actions in the future. Currently, LAVs appear as the most feasible vaccine choice in the immediate future, but subunit vaccines are likely the long-term alternative, especially for ASF-free areas reluctant to implement LAVs in the field [9,44,45]. Nonetheless, the little information available regarding the ASFV antigens involved in protection is a major gap for the short-term development of efficient subunit vaccines.

It has long been known that both antibodies [19,20] and CD8^+^ T-cells [21] can play important roles in the protection afforded by LAVs, however, little information exists about the antigens involved in such protection. In fact, the experimental subunit vaccine prototypes so far tested, have demonstrated little or non-reproducible protection [22,46,47,48,49]. To complicate the picture, the protection afforded by the structural proteins p54, p30, p72, and CD2v, initially identified as protective humoral determinants in genotype I strains [50,51,52], failed to cross-protect against other ASFV strains [49,53], including genotype II Georgia2007/1 [47,48,54].

Work performed in our laboratory using DNA immunization as a tool and ubiquitin as a genetic adjuvant to improve SLA I presentation clearly demonstrated the ability of specific ASFV antigens to protect against E75 (genotype I strain) lethal challenge in the absence of detectable antibodies [22,23]. The protection afforded in one of these studies correlated with the induction of specific CD8^+^ T-cells against two epitopes from the CD2v antigen [22]. Unfortunately, the protection observed was not reproducible against Georgia2007/1 [24], confirming studies published by others [47,54]. Variability of the CD2v sequence among ASFV isolates [55] might account for the lack of protection against Georgia2007/1. Thus, the two CTL epitopes described in E75 are not present in the Georgia2007/1 CD2v sequence. Furthermore, the rest of the ASFV antigens described as potentially protective against genotype I viruses have failed to protect against Georgia2007/1, despite being highly conserved, perhaps indicating differences in the pathogenesis and/or in the capabilities to interfere antigen presentation pathways between the ASFV strains. With this data at hand, identification of protective antigens in other ASFV strains, such as the promising results recently described using genotype I OURT strains [48,56], would need to be confirmed in Georgia2007/1 if willing to fight this ASFV strain. Here, we provide experimental evidences demonstrating the presence of at least three CD8^+^ T-cell ASFV determinants promiscuously recognized by pigs surviving Georgia2007/1 challenge, providing the bases for future antigen discovery.

The low rate of success of our peptide prediction approach, with only one peptide out of the 330 theoretical best peptides identified by specific CD8^+^ T-cells, is in consonance with previous results from the lab [57]. Due to the concerns that we had when performing the in silico predictions based on a single prediction tool, we decided to perform a multiparametric analysis not only based on NetMHCpan. Despite novel immunoinformatics tools have been recently developed [58], at the time of the analysis little data was available from swine [59,60,61,62], which forced to complete our multiparametric prediction tool with experimental data obtained mainly from human and mouse studies. Most probably due to the imperfections of our predictive methodology, our approach has allowed the identification of only one promiscuously recognized ASFV T-cell determinant within the Georgia2007/1 MGF100-1L protein. Being aware that other methods might provide better results, the reality is that up to today little reports are available providing definitive information about potential promiscuous vaccine targets against ASFV. As a good example, recent work performed in our own laboratories [63], made it possible to predict a completely different set of potential ASFV T-cell epitopes, and only one partially overlapped with the ones here described. Interestingly, the two overlapping SLA I-binding peptides found in our proteomic studies, partially overlapped with a longer polypeptide predicted as a potential CD4^+^ T-cell peptide within the major structural ASFV p72 protein (encoded by the B646L ORF) but, so far, no experimental work has confirmed its recognition by ASFV-specific T-cells. The work here presented just add a little bit of knowledge to a very complex issue that should be taken seriously if willing to obtain safer subunit vaccines into the market. Further work should be invested to optimize both the computational searches and the identification of optimal vaccine targets for the future. Even though the ranking criteria established was based on scientific grounds, limiting the selection to the best 330 peptides was not absent of risks, particularly when Georgia2007/1 encodes 166 ORFs and taking into account the SLA I heterogeneity existing in the domestic pig population. It should be pointed out that none of the predicted peptides tested matched the ones identified in the immunopeptidomic studies. Still, the newly discovered MGF110-1L peptide was promiscuously presented by APCs, despite its length (19 amino acids), theoretically suboptimal for SLAI binding, supposedly by directly fitting into the binding groove with a bulged conformation or by being internalized and processed to the contained overlapping peptides [64,65].

On the other hand, immunopeptidomics has been demonstrated as a powerful tool to unmask CD8^+^ T-cell epitopes, confirming previous studies with other pathogens [66,67,68,69]. SLA I immunoprecipitation studies performed on cell extracts from PAMs infected with the live attenuated BA71ΔCD2 ASFV and the parental BA71 isolate allowed the identification of five (out of the 137 SLA I bound peptides profiled) as novel ASFV-specific T-cell epitopes, three of them recognized by more than one pig. The ratio of success obtained fits with the fact that only a minor fraction of a large number of potential candidates in the MHC-peptide repertoire induce a specific immune response [70]. However, none of the identified SLA I-bound peptides should be discarded, since they might be real CTL peptides associated to SLA I alleles not present in the animals tested, since our screening was performed in outbred commercial pigs. Additionally, the immunodominance phenomenon might also prevent the identification subdominant epitopes that might be relevant in protection [29]. Confirming this hypothesis, pig immunization studies performed with cocktails of synthetic peptides, identified in the immunopeptidomics assays and grouped according to their theoretically binding affinity for SLA I as strong and week binders, induced promiscuous CD8^+^ T-cell responses that in vitro recognized not only the peptides used in vivo, but also ASFV (data not shown). The fact that these peptides were not recognized by PBMCs of surviving pigs, but were capable of in vivo stimulating T-cells that recognize ASFV in vitro, opens the possibility to explore the protective potential of subdominant epitopes, otherwise masked due to the immunodominance phenomenon, as it has been described before for other infectious agents [29]. Moreover, potential CTL epitopes might have escaped our read-out assay. Thus, our assay was limited to the detection of the T-cell repertoire present in the blood at a given time and capable to stimulate a response, while different repertoires might be present in primary ASFV target organs, lymphoid tissue, at different times post-infection and/or with other effector characteristics.

Finally, the peptide repertoire identified might be biased by the effector and target cells used in the assays. Despite macrophages being the main ASFV target [71,72], epithelial cells and dendritic cells (DCs), might also interact with ASFV [15,18,73,74,75,76]. Alternative antigen processing pathways exclusively present in DCs might render different peptide repertoires to the specific CD8^+^ T-cells [77], an option that should be tested in the future. Moreover, the choice of effector cells for the assay will also define the fate of the experiments. Since Georgia2007/1 is a highly virulent and lethal ASFV strain, experimentally infected pigs die before having the chance to develop ASFV-specific T-cells. To circumvent this problem, before the Georgia2007/1 challenge, pigs were immunized with BA71∆CD2, a recombinant LAV lacking CD2v, capable to confer homologous and heterologous protection, including against Georgia2007/1 [12]. In vivo cross-protection seemed to correlate with the ability of immunized pigs to induce specific CD8^+^ T-cells capable to recognize homologous and heterologous ASFV strains [12]. Despite the limitations derived from the low number of replicates, it seems that BA71∆CD2 is presented in the SLA I-restricted pathway quantitatively and qualitatively better than its parental virulent BA71 strain, which might explain at least partially the improved CD8^+^ T-cell responses induced in vivo. We are currently extending our antigen-presentation study to Geogia2007-infected cells, albeit preliminary studies do not evidence significant differences to that obtained with BA71-infected cells and again seems to be quantitatively less efficient than BA71ΔCD2-infected cells at presenting SLA I-peptides (data not shown).

The presence of out of frame SLA I-binding peptides in PAMs infected with BA71ΔCD2 might demonstrate once again its superior presentation in the SLA I-restricted pathway. The presence of out of frame SLA I-peptides have been described both in tumors [78,79,80] and virus infections [81,82,83], including in ASFV-infected cells [84]. As before mentioned, their protective relevance should not be ruled out despite none being recognized by PBMCs from recovered pigs, since they might have relevant functions during ASFV infection and protection. Previous results showed that CD2v suppresses mitogen-dependent lymphocyte proliferation in vitro [85], most probably through its C-terminal end, located in the cytoplasm of the infected cell. So far, the C-terminal end of CD2v has been demonstrated to interact with multiple immune mediators, including the SH3P7 [86] and AP-1 [87], involved in key aspects of cell trafficking, and the latter being involved in SLA I downregulation during HIV infection [88,89]. Future efforts will be done to understand the intrinsic mechanisms by which the depletion of CD2v from BA71 yields an attenuated ASFV strain (BA71∆CD2), capable of improving antigen presentation in vitro and inducing efficient and cross-protective immune responses in vivo.

While virus infections usually lead to the development of a host response against a narrow range of dominant peptides, expression of antigens in fusion with ubiquitin has been previously described to promote CD8^+^ T-cell responses [22,23], and as a screening tool to identify dominant and subdominant epitopes [29,90]. Thus, the use of transfected fibroblasts as APCs in the screening methodology of the present work has proven a valuable strategy to monitor the immune response of the pigs ex vivo. A similar screening of transiently transfected APCs with immune cells from convalescent animals as effector cells was previously described for the identification of CD8^+^ T-cell antigens in other models [91,92], as well as in ASFV using random cDNA clones [84]. Despite fibroblasts not being susceptible to infection with virulent ASFV strains, they are perfectly capable of presenting antigens in the SLA I context to specific CD8^+^ T-cells [93]. This, together with the feasibility of establishing fibroblast cell lines from individual animals that are highly susceptible to DNA transfection, defined them as optimal tools to identify ASFV-full length proteins containing CD8^+^ T-cell epitopes. The identification of MGF110-1L, A238L and MGF505-7R as promiscuous ASFV antigens confirmed the suitability of the methodology, taking into account that peptides from these same proteins were independently defined as ASFV-specific CD8^+^ T-cell determinants overall, albeit recognized by a smaller percentage of animals than the correspondent full-length protein. Altogether, the results obtained indicate that the methods here employed might validate each other, since the same ASFV determinants were identified as inductors of CD8^+^ T-cell responses. Future experiments should determine the protective potential of these newly described T-cell determinants and, if so, the specific mechanisms by which they confer such protection, including working with PBMCs deprived of CD4^+^ T-cells to definitively confirm their identity. Enough evidences seem to confirm the T-cells here described as ASFV-specific CD8^+^ T-cells: (1) the predictions made were based on potential SLA I overlapping peptides; (2) all peptides described by proteomics were selected, due to the immunoprecipitation of SLA-peptide complexes using specifically binding to anti-SLA I antibodies; (3) the ASFV full-length proteins described as T-cell determinants were identified by using transfected fibroblasts that express SLA I, but not SLA II molecules on their surface; (4) Figure S2, defines as single positive CD8 T-cells the T-cell subset, recognizing the ASFV-specific peptide mix used, while a stimulation with BA71∆CD2 stimulated all three T-cell subsets: single positive CD8 T-cells, single positive CD4 T-cells and double positive CD4 CD8 T-cells, corresponding with CTL, T-helper and memory T-cells, respectively.

Selection of the optimal protective antigens is crucial for the success of future ASFV subunit-based immunization approaches. Moreover, characterization of conserved antigens might be key to develop cross-protective vaccines, an important feature for endemic regions as Africa, where many ASFV strains have been described to be concomitantly circulating [94,95]. Future efforts should be directed at identifying promiscuous and cross-protective ASFV antigens, as well as the optimal delivery methods and/or the appropriate adjuvants to be used in the field, a critical point when the cost is crucial for the success of the vaccine.

## Figures and Tables

**Table 1:** Predicted epitopes within the peptide MGF100-1L_68–86_ (LQMAPGGSYFITDNMTEEF). The table summarizes the number of predictions that overlap with each sequence and the SLA I alleles predicted to present it.

68	69	70	71	72	73	74	75	76	77	78	79	80	81	82	83	84	85	86	Overlapping Peptides
**L**	**Q**	**M**	**A**	**P**	**G**	**G**	**S**	**Y**											8
SLA-1*0801, SLA-2*1001																			
**L**	**Q**	**M**	**A**	**P**	**G**	**G**	**S**	**Y**	**F**										9
SLA-1*0801, SLA-2*0601, SLA-2*1001																			
**L**	**Q**	**M**	**A**	**P**	**G**	**G**	**S**	**Y**	**F**	**I**									**12**
SLA-2*0601, SLA-2*1001																			
	**Q**	**M**	**A**	**P**	**G**	**G**	**S**	**Y**											8
	SLA-1*0201, SLA-1*0202, SLA-1*0401, SLA-1*0701, SLA-1*0702, SLA-1*0801, SLA-1*1301, SLA-2*1001																		
	**Q**	**M**	**A**	**P**	**G**	**G**	**S**	**Y**	**F**										9
	SLA-1*0201, SLA-1*0202, SLA-1*0401, SLA-1*0801, SLA-1*1301, SLA-2*1001																		
		**M**	**A**	**P**	**G**	**G**	**S**	**Y**	**F**	**I**									**12**
		SLA-2*0502																	
								**Y**	**F**	**I**	**T**	**D**	**N**	**M**	**T**	**E**	**E**	**F**	**12**
								SLA-1*1301											
									**F**	**I**	**T**	**D**	**N**	**M**	**T**	**E**	**E**	**F**	9
									SLA-1*1301										
										**I**	**T**	**D**	**N**	**M**	**T**	**E**	**E**	**F**	6
							SLA-1*0201, SLA-1*0401, SLA-1*0601, SLA-1*1301												

**Table 2:** Summary of SLA I-restricted ASFV peptides identified by MS-based immunopeptidomics.

Function/Protein	Total Peptides	BA71	BA71ΔCD2	Activity/Similarity	Temporal Expression
**Multigene Families**	**14**	**5**	**9**		
MGF110-6L	2	1	1		Early
MGF360-10L	2	1	1		Unknown
MGF360-8L	2	1	1		Early
MGF505-1R	3	1	2		Early
MGF505-2R	1	0	1		Late
MGF505-3R	1	0	1		Early
MGF505-5R	1	0	1		Early
MGF505-7R	1	0	1		Early
MGF505-9R	1	1	0		Early
**Transcription, Replication and Repair**	**41**	**13**	**28**		
C315R	1	0	1	Transcription factor IIB-like	Early/Late
C475L	4	1	3	Poly(A) polymerase large subunit	Late
D1133L	7	3	4	Helicase superfamily II	Late
D205R	3	0	3	RNA polymerase subunit 5	Early
D250R	1	0	1	Ribonucleotide reductase (small subunit)	Early
D339L	1	0	1	RNA polymerase subunit 7	Early
E301R	2	1	1	Proliferating cell nuclear antigen-like protein	Late
EP1242L	2	2	0	RNA polymerase subunit 2	Early/Late
EP364R	1	0	1	ERCC nuclease domain	Late
EP424R	3	2	1	FtsJ-like methyl transferase domain	Early
F334L	1	0	1	Ribonucleotide reductase (small subunit)	Early
G1211R	4	1	3	DNA polymerase family B	Early/Late
G1340L	1	0	1	VV A8L-like transcription factor	Late
H359L	1	1	0	RNA polymerase subunit 3	Early
I243L	1	1	0	Transcription factor SII	Early/Late
M448R	1	0	1	RNA ligase	Late
NP1450L	1	0	1	RNA polymerase subunit 1	Early/Late
NP419L	1	0	1	DNA ligase	Early/Late
P1192R	4	1	3	DNA topoisomerase type II	Early/Late
Q706L	1	0	1	Helicase superfamily II	Late
**Morphogenesis**	**24**	**12**	**12**		
A137R	1	0	1	Protein p11.5	Late
A151R	1	0	1	Protein oxidation pathway	Early/Late
B602L	3	1	2	Chaperone	Late
B646L	5	2	3	Major capsid protein p72	Late
CP2475L	10	7	3	Polyprotein pp220	Late
E120R	1	0	1	Structural protein p14.5, DNA-binding protein	Late
E183L	2	1	1	Structural protein p54	Late
E248R	1	1	0	Structural protein	Early/Late
**Host Cell Interaction**	**6**	**1**	**5**		
A179L	2	1	1	Bcl-2 apoptosis inhibitor	Late
A238L	1	0	1	IkB-like protein, inhibitor of host gene transcription	Early
QP383R	3	0	3	Nif-S like	Late
**Uncharacterized**	**47**	**19**	**28**		
B117L	1	0	1		Late
B125R	2	0	2		Late
B475L	9	5	4		Late
C129R	3	1	2		Late
C257L	1	0	1		Late
CP123L	2	0	2		Late
CP312R	1	0	1		Early/Late
DP238L	2	1	1		Early
E111R	1	0	1		Early/Late
F317L	3	1	2		Late
H233R	3	1	2		Late
H339R	3	1	2		Late
I226R	2	2	0		Early/Late
I73R	2	1	1		Early/Late
I9R	1	0	1		Unknown
K145R	6	3	3		Late
M1249L	5	3	2		Late
**TOTAL**	**132**	**50**	**82**		

**Table 3:** ASFV epitopes identified by immunopeptidomics that are recognized by specific T-cells from BA71ΔCD2-vaccinated pigs that survived the lethal Georgia2007/1 challenge.

Peptide Sequence	Protein	Responding Animals	Sample	Georgia2007/1 Identity
NPTIIMEQY	H339R	1/10 (10%)	BA71	100%
KNILNTLMF	I226R	1/10 (10%)	BA71	100%
DKDGNSALHYL	A238L	6/20 (30%)	BA71ΔCD2	100%
AKIVEEGGEES	K145R	4/20 (20%)	BA71/BA71ΔCD2	100%
NSTLVIRI	MGF505-7R	4/20 (20%)	BA71ΔCD2	87.5% (NSTLVIRL)

**Table 4:** E75 ASFV gene expression library encoding each open reading frame (ORF) with the ubiquitin gene at the N-terminus and under the control of the CMV promoter.

Plasmid Mix	E75 Locus	Protein Name	Plasmid Mix	E75 Locus	Protein Name
Mix 1	2	DP93R	Mix 5	92	B407L
	3	MGF360-2L		93	B175L
	4	KP177R		94	B263R
	6	L60L		104	O174L
	7	MGF360-3L		109	D250R
	8	MGF110-1L		110	D129L
	10	MGF110-13L		111	D79L
	13	MGF110-12L		116	D345L
	14	MGF110-14L		117	S183
	15	MGF360-4L		118	S273R
Mix 2	16	MGF360-6L		120	H359L
	17	X69R	Mix 6	121	H171R
	18	MGF300-1L		122	H124R
	20	MGF300-2R		132	E184L
	22	MGF300-4L		133	E183L
	23	MGF360-8L		135	E301R
	25	MGF360-10L		137	E199L
	26	MGF360-11L		138	E165R
	28	MGF360-12L		139	E248R
	30	MGF360-14L		140	E120R
Mix 3	31	MGF505-2R		141	E296R
	33	MGF505-4R		142	E111R
	35	MGF505-6R	Mix 7	143	E66L
	36	MGF505-7R		144	I267L
	39	A224L		151	I215L
	40	A104R		152	I177L
	41	A118R		153	I196L
	45	MGF360-15R		158	MGF100-2L
	46	A238L		161	I8L
	47	A859L		163	I10L
Mix 4	48	A179L		164	L11L
	49	A137R		167	DP96R
	50	F317L		168	MGF360-19R
	53	F165R			
	55	K205R			
	56	K78R			
	63	EP152R			
	66	EP364R			
	77	C62L			
	83	B438L			

## Data Availability

Data is contained within the article and supplementary material.

## Supplementary Materials

The following are available online at https://www.mdpi.com/2076-393X/9/1/29/s1, Table S1: In silico predictions selected for the presence of multiple predicted CD8^+^ T-cell epitopes in a given polypeptide; Table S2: ASFV proteins from which peptides were selected by in silico predictions; Table S3: SLA I-restricted peptides identified by MS-based immunopeptidomics; Figure S1: Complete view of the western blot (WB) corresponding to Figure 1D; Figure S2: Intracellular IFNϒ staining of ASFV peptide-specific T cells.
