# Supplementary material for: Identification of Promiscuous African Swine Fever Virus T-Cell Determinants Using a Multiple Technical Approach
Source: Vaccines (Basel). 2021 Jan 7;9(1):29. doi: 10.3390/vaccines9010029 (PMC7825812; doi:10.3390/vaccines9010029)

**Supplementary Figure S1**. Complete view of the western blot (WB) corresponding to Figure 1D showing the detection of SLA I molecules in supernatants (SN) of infected cell lysates after incubation with anti-SLA I-coupled sepharose beads, in the last sepharose wash before elution (Wash), or in the eluted SLA I-peptide complexes (Elution). M: Molecular weight marker (mouse anti-His tag HRP-conjugated (Novex) 1:100000). A Fluorchem HD2 (Alpha Innotech) was used for imaging.


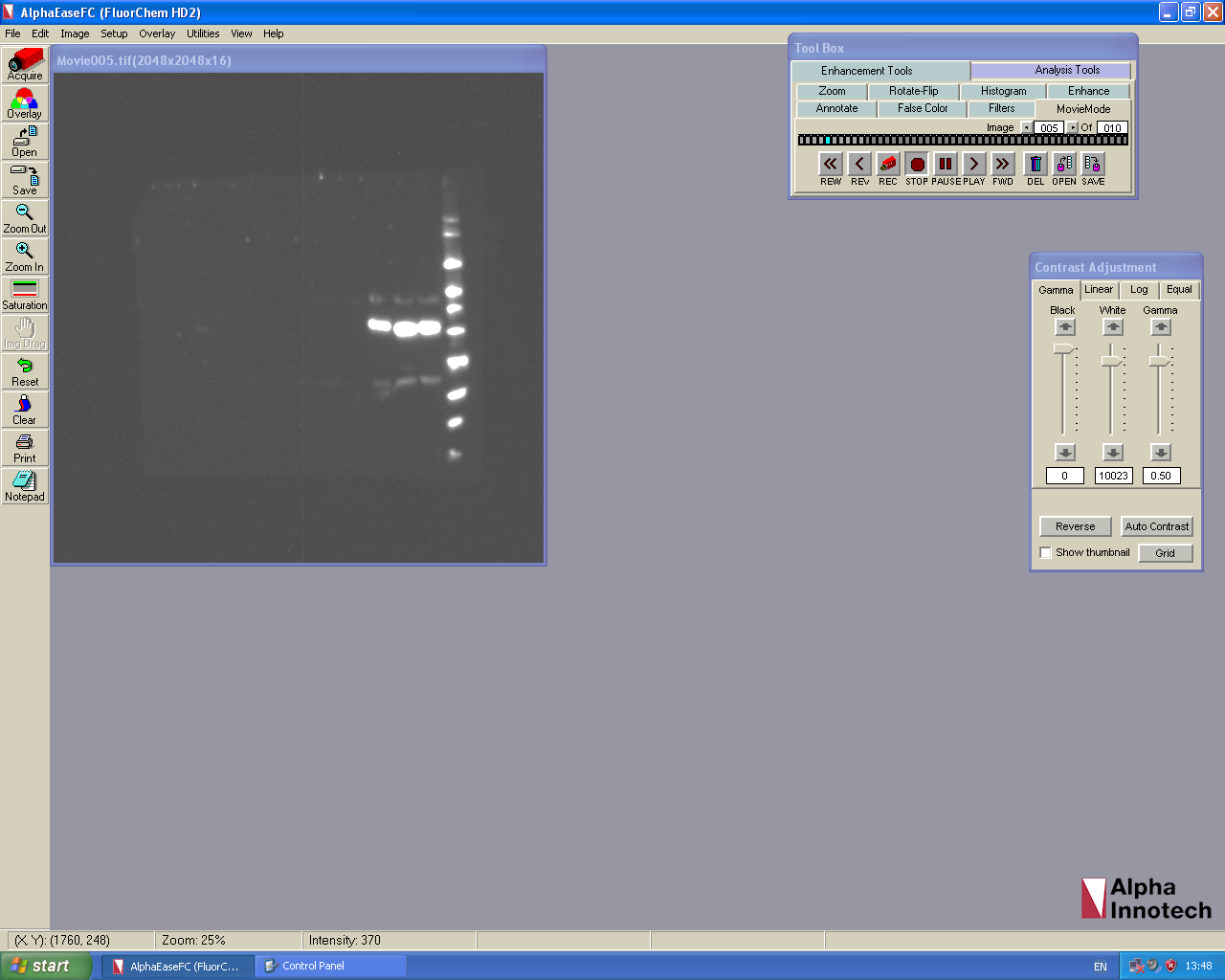


**kDa**

**160**

**80**

**60**

**120**

**50**

**40**

**30**

**20**

**15**

**10**

**Mock**

**BA71**

**BA71ΔCD2**

**Mock**

**BA71**

**BA71ΔCD2**

**Mock**

**BA71**

**BA71ΔCD2**

**SN**

**Wash**

**Elution**

**M**

**Supplementary Figure S2.** Intracellular IFNγ staining of ASFV peptide-specific T cells. PBMCs from (A) a representative BA71ΔCD2-immunized and (B) a control animal stimulated for six hours with a mix of previously identified ASFV-specific peptides (ELISpot results) or BA71ΔCD2, and then incubated for two additional hours with Brefeldin A (GolgiPlug, BD Biosciences) to allow the intracellular accumulation of IFNγ. After stimulation, cells were stained with LIVE/DEAD Fixable Red Dead Cell Stain (Invitrogen, 1/1000), anti-CD3 PE-Cy7 (BD Pharmingen, BB23-8E6-8C8, 1/100), anti-CD8a FITC (BD Pharmingen, 76-2-11, 1/50), anti-CD4a PerCP-Cy5.5 (BD Pharmingen, 74-12-4, 1/100), and anti-γδTCR APC (BD Pharmingen, MAC320, 1/100). Afterwards, cells were fixed with BD Cytofix/Cytoperm, and anti-IFNγ PE (BD, P2G10, 1/80) was incubated in BD Perm/Wash buffer. A BD FACSAria II (BD Biosciences) was used for analysis. Cells were gated on single cells, live lymphocytes, CD3+γδTCR-, and CD4-CD8+, CD4+CD8+ or CD4+CD8-.

**CD8+**

**Mock**

**BA71ΔCD2**

**Peptides**

**CD4+CD8+**

**CD4+**

**IFNγ**

**BA71ΔCD2-IMMUNIZED ANIMAL**

**A**


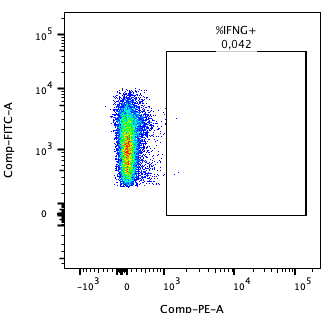

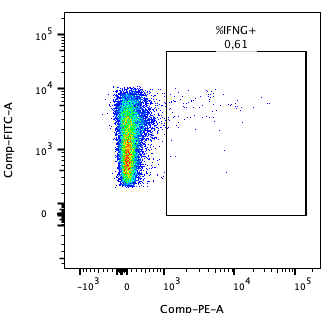

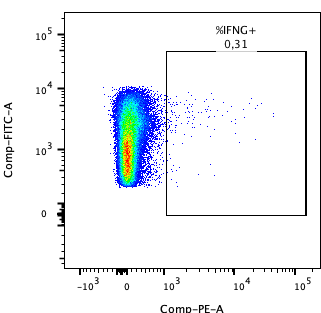

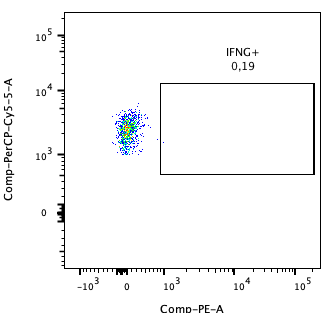

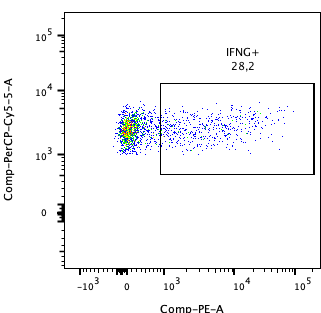

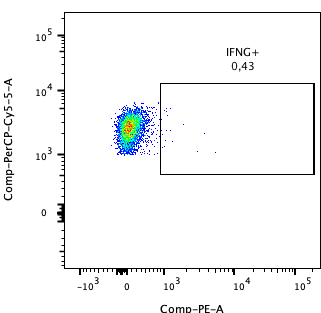

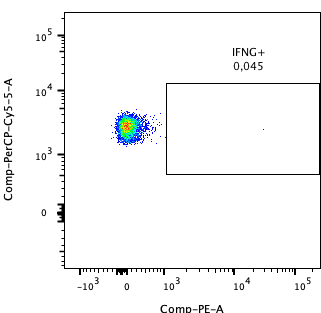

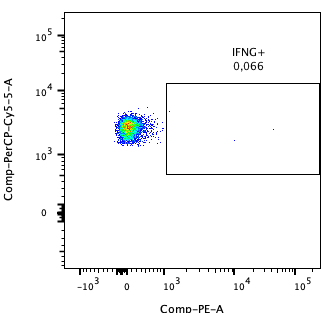

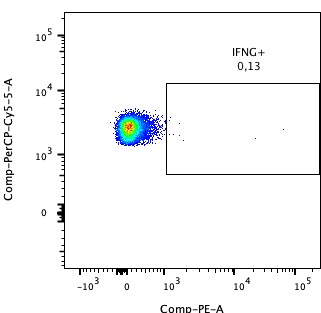


**CD8+**

**Mock**

**BA71ΔCD2**

**Peptides**

**CD4+CD8+**

**CD4+**

**IFNγ**

**CONTROL ANIMAL**

**B**


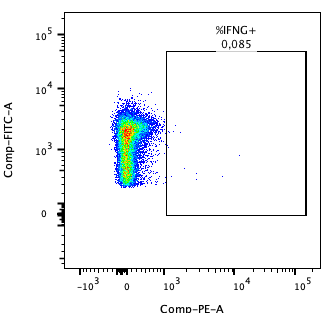

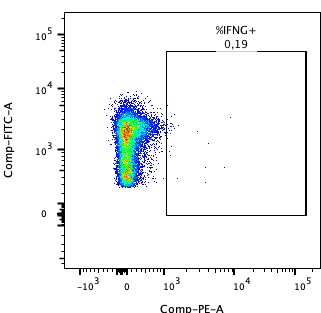

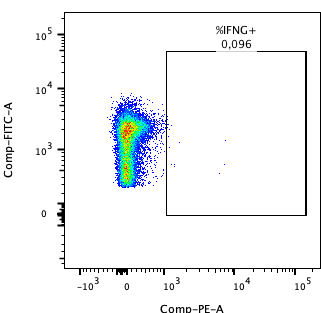

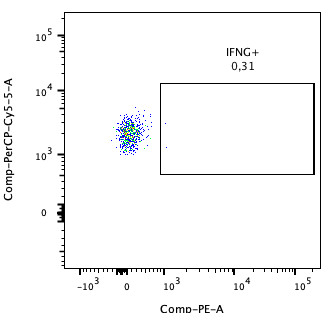

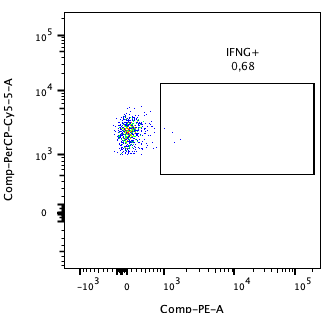

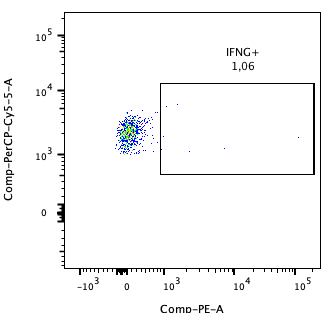

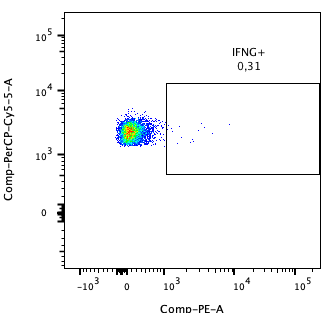

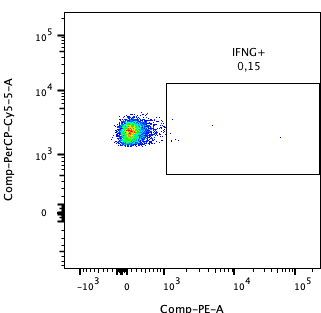

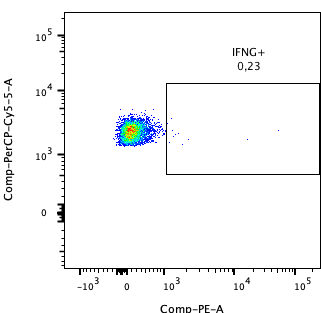

Supplement: Supplementary file 1 [file vaccines-09-00029-s001.zip › Sup/vaccines-1034457-sup-3rd revision.docx]
